# Supplementary material for: Evaluation of content validity and feasibility of the eVISualisation of physical activity and pain (eVIS) intervention for patients with chronic pain participating in interdisciplinary pain rehabilitation programs
Source: PLoS One. 2023 Mar 10;18(3):e0282780. doi: 10.1371/journal.pone.0282780 (PMC10004540; doi:10.1371/journal.pone.0282780)
Supplement: Attachment — Submitted filename: Response to Reviewers.docx [file pone.0282780.s001.docx]

**Editor’s and Reviewer Comments - point-by-point response**.
Please note that indicated pages and rows, in the column Action, refer to the revised paper without tracked changes, the file labeled 'Manuscript'.

| **Editor’s Comments** | **Author Response** | **Action** |
| --- | --- | --- |
| 1. Please ensure that your manuscript meets PLOS ONE's style requirements, including those for file naming. | Thank you. We have checked the PLOS ONE’s style requirements once more and believe we now have formatted correctly. | Revision of file naming. |
| 2. We noted in your submission details that a portion of your manuscript may have been presented or published elsewhere. “No, except from a recent poster presentation at the Conference: 12th Congress of the European Pain Federation EFIC, Dublin, Ireland, 27-30 April 2022, entitled: "Content Validity and Feasibility of the eVISualisation of physical activity and pain (eVIS) intervention in Interdisciplinary Pain Rehabilitation Programs: Valuable steps in a systematical development and evaluation process". Figures and data are not identical with our submitted manuscript to PLOS.”  Please clarify whether this conference proceeding was peer-reviewed and formally published. If this work was previously peer-reviewed and published, in the cover letter please provide the reason that this work does not constitute dual publication and should be included in the current manuscript. | To clarify, the submitted abstract was peer-reviewed by the Conference’s Scientific Programme Committee but not formally published. The poster, which was neither peer-reviewed nor formally published, depicted the study process and conclusion but includes no quantitative or qualitative data. This conference proceeding does not constitute dual publication of the paper.  During our scrutiny of this query we realized that the concept figure of eVIS was previously published in our study protocol - and therefore we have now attributed Figure 2 to the source reference and CC license, CC BY-NC 4.0. | Commented in Cover letter. |
| 3. We note that you have indicated that data from this study are available upon request. PLOS only allows data to be available upon request if there are legal or ethical restrictions on sharing data publicly. For more information on unacceptable data access restrictions, please see <http://journals.plos.org/plosone/s/data-availability#loc-unacceptable-dataaccess-restrictions>.  In your revised cover letter, please address the following prompts:  a) If there are ethical or legal restrictions on sharing a de-identified data set, please explain them in detail (e.g., data contain potentially sensitive information, data are owned by a third-party organization, etc.) and who has imposed them (e.g., an ethics committee).  Please also provide contact information for a data access committee, ethics committee, or other institutional body to which data requests may be sent. | Thank you for pointing out this ambiguity in our statement.  Our reply to the Data availability query has now been changed to only a **'No'** and we have revised the Data Availability Statement for better clarity on the ethical restrictions imposed by the ethics committee and legal restrictions. Moreover, we added contact information to institutional body, complementary to principal investigator.  ***Revised Data Availability Statement*:**  Data cannot be shared publicly because of ethical restrictions involving human research participant information.  Data contains potentially identifying information and sensitive patient information. Data are collected from a small group of participants and especially qualitative data, such as free-text comments in questionnaires and diary notes, risk compromise participant privacy.  Data is only available from the Dalarna University Institutional Data Access for researchers who meet the criteria for access to confidential data, after approval from the principal investigator. Data requests may be sent to the Dalarna University Institutional Data Access [dataskydd@du.se](mailto:dataskydd@du.se).  De-identified individual data (*participant ratings*) used in this publication will be made available on reasonable request to the principal investigator. However, such a request must lie within the limits of the ethical approval for this project. In addition, to uphold data safety and other legal aspects, relevant agreements must be established prior to data being available outside the research group. | -Revision of Data Availability Statement.  -Comment in Cover letter.  November 18, 2022  Additional comment in response to requested clarification:  Yes, data requests can be sent to the Dalarna University Institutional Data Access (dataskydd@du.se), directly  We have now revised the statement- marked with yellow highlight. |
| **Reviewer #1:** | **Author Response** | **Action** |
| The content of the abstract is adequate, making a brief presentation of the introduction, summarising the most important aspects of the method, highlighting the most relevant results and conclusions. However, I would recommend the authors not to include concepts used in the title and/or abstract in the keywords, thus increasing the chances of being listed in search engines | Thank you for pointing this out.  We have now rephrased three of our key words to increase our visibility in search engines; Content validity and Feasibility have been replaced by **Evaluation study** and Physical activity with **Exercise**. | Page 3, rows 48-49 |
| Indeed, chronic musculoskeletal pain is a complex disease, often used generically when the exact causes of the pain are not known, encompassing many types of pain, so its definition is not simple. The authors could make some reference to the current definition of chronic musculoskeletal pain, its possible causes and whether the origin could modify the intensities and effects. | Thank you for this recommendation.  In the Introduction, we have now chosen to provide some clearer distinctions between chronic pain, and the recent advances in classifications relating to these. | Page 4, row 54-60 |
| The rest of the introduction is well directed towards the objectives of the research, although it would be advisable to include what the hypothesis was. | Thank you for this suggestion. We have now elaborated on our hypothesis in the Introduction and Discussion. | Page 6, row 115-116  Page 32, row 615-618 |
| The methodology is well described, but I have some doubts regarding the selection of participants, although I do not feel competent to assess whether the number of participants and their selection was adequate. | Thank you. We have now revised the methods section regarding participant selection and also commented on this query in the discussion. | Page 9-11  Page 30, row 572-584  Page 31, row 604-609  Page 35, row 701-708 |
| The results section is clearly developed, with sufficient information on all findings, including tables that facilitate the understanding of the quantitative data, as well as explanations of the qualitative aspects. | Thank you | No action |
| The discussion presents the main findings and debate on the encounters with classical and current references. | Thank you | No action |
| **Reviewer #2:** | **Author Response** | **Action** |
| Thank you for the opportunity to review this manuscript. I would like to congratulate the authors on their work and for their contribution to the scientific literature. Their work is important and relevant to optimize the health of patient living with chronic pain.  However, I have highlighted several aspects that need clarifications, along with comments and major preoccupations concerning their paper, which I feel the authors should consider. | Thank you | No action |
| **Introduction**:  - The problematic is well described, and its relevance is well put forward by the authors  - The rationale for the proposed intervention is clear. A brief description is present which, in my opinion, lacks details (i.e., mechanisms of action). Yet, it could still be considered adequate for the introduction section.  - A theoretical framework is highlighted. | Thank you. We have now built on the rationale a little earlier in the text, and condensed the descriptions of behavioral change techniques, with referral to theory on proposed mechanisms of action | Page 4, row 72-73  Page 5, row 81-87 |
| **Objectives**:  - We believe that the formulation of the objectives is misleading and could be improved.  After reading the methods section, there is clearly more than 1 objective contained in the paper, but the formulation does not reflect that. Thus, it should be divided into 2 (i.e., content validity and feasibility) or even 3 distinct objectives since the methods for each objective vary greatly. This issue has great repercussion in the readability of the overall paper and particularly the methods section (see comments below). | We have now made revisions according to your suggestion.  We have rephrased the objectives and divided them into two:   1) To evaluate the content validity of eVIS in a pre-clinical context 2) To evaluate the content validity and feasibility of eVIS in a clinical context | Page 6, Row 120-123. |
| **Methods**:  - For better readability, we invite the authors to consider fragmenting their methods by objective (i.e., step 1 and 2) as each objective is attained with a specific method. | Thank you for this suggestion. We have now restructured the Methods section and fragmented our methods by objective, indicated by Step 1 and Step 2 headings, in accordance with our objectives. Steps 1 and 2 are further detailed under Study design and context.  As a consequence, we also moved the description of the eVIS intervention up in Methods, under the heading Subject of evaluation, before the detailing of the two methods. | Page 6 to 18:  -Consequent headings in accordance with two objectives.  -Page 7, 135-141  -Transfer of the description of the eVIS intervention, from page 10 to 8.  -Revision Fig 1 |
| - Table 1 provides very little information on the participants. Were other characteristics of the sample collected other than sex? We suggest to the authors to provide additional information on the participants of the study if they have such data.  Otherwise, revise (major) Table 1.  Moreover, information regarding participants represents a result, which is usually presented in the results section. | Thank you for pointing this out.  In Step 1 and the questionnaire used for Assessment 1, we included a few questions on characteristics to which participants were prompted depending on participant category (patient, clinician, or researcher).  These were mainly intended to establish participants’ relevant topic expertise, that they were representative samples of the content domain. We did not collect any additional information.  We have now provided some more information on the participants and reordered text to give a more coherent description of their expertise, as part of our methods section.  We also moved Table 1 to Results section, for an overview of included subjects in the whole study. | Page 9-10, 194-236  Page 18-19, Table 1 |
| - Recruitment of the study participants for step 1 lacks details. How were the subject expert recruited? (line 146) How was “relevant knowledge” defined? (line 141). The authors need to make it explicit – how were the potential participants deemed/considered as experts?  What were the criteria? | Thank you, we agree the eligibility criteria might be perceived as too vague. We have now elaborated more on our descriptions of what constitutes subject expertise, and how we approached the recruitment process to attain suitable participants on the basis of their experience of the phenomenon of interest.  A strategic recruitment of study participants was applied in step 1 to achieve a broad representation of the eVIS target population, combined with documented expertise either clinical or research-based within the fields relating to eHealth, physical activity, behavior change, outcome measures etc. The expertise area was verified by the initial questions in the questionnaire used for Assessment 1.  From our recruitment process, we were confident that subjects were representative samples of the content domain. | Page 9-11, row 184-236 |
| - Recruitment process needs further description. Currently, the risk of selection bias seems highly likely based on the details contained in the paper. | We have now elaborated more on how the recruitment was done, by adjoining text sections into more coherent parts and complemented with details. | Page 9-10, row 184-211 |
| - Same issue related to the recruitment process of the study participants is present for step 2. | We have now described the recruitment process in step 2 in more detail. | Page 10-11, row 214-232 |
| - Why only 2 physiotherapists were used as subject experts for follow-up interview? (line 177-8)? Another selection bias? Only 2 PT is enough to provide solid data for this objective? | All three physiotherapists were invited to the follow-up interview. However, one was unable to participate, due of staff restrictions due to the pandemic. We have now added the reason for one missing in the text.  As our follow-up interview mainly concerned feasibility aspects of the Start-up process, on how to best accommodate needs from the clinic to make initiation and patient recruitment as easy as possible, we believe the discussions provided enough input for our following revisions. | Page 11, row 227-228 |
| - The intervention is well described.  However, it would be relevant to have more information on its mechanism of action, and more specifically on the communication part.  How is the information gathered via the eVIS intervention used by the health professionals who work within the IPRP context?  Also, it would be interesting to provide additional information on how the documentation and visualisation parts of the eVIS intervention translate into the communication part. | We briefly address the potential mechanisms of action in the Introduction section. These were not explicitly in the scope of this study.  The information gathered via the eVIS intervention is intended to be communicated between caregivers and patients in in synchronous one to one meeting, that is, the patient logs in and shows the documentation.  There is no formalised directive on how this communication is to be implemented, it is up to the caregiver and patient to use and integrate the clinical tool as fits them within the ordinary care. | 1.Page 5  2. Page 8, row 174  3. Page 9, row 177-178 |
| - As for the content Validity Index (CVI), there is no specific link to the objective and how it was operationalized | Thank you, we understand the rationale for using the CVI as indicator of content validity was missing. We have now tried to clarify how the CVI fits into the process of judgment quantification by adding text in two different places in the manuscript, under ‘Study design and context’ and ‘Outcomes’. | Page 7, row 128-134  Page 12, row 257-259  Page 12-13, row 273-275 |
| - The assessment rounds for the consensus process in step 1 is hard to follow. It lacks details on how the consensus experts worked together and which changes were applied based on the consecutive rounds.  What were the criteria to make such changes?  Was the consensus process standardized in some way?  How were the information analyzed by the subject experts? | Thank you  An introduction on the consensus panel task has now been added under ‘Study design and context’ as well as a more detailed explication of the consensus process in step 1, under ‘Data collection and assessment processes’. | Page 7, row 131-134  Page 11-12, 239-253  Page 14, row 315-320 |
| - The assessment of the qualitative data from the questionnaires (open question), interviews and journaling are poorly described. No mentions of specific data analysis methods (i.e., coding, multiple evaluators, themes, software used?). No mentions of an analysis framework. | We have now clarified that no specific qualitative analysis framework/ methodology was applied in the interpretation of the free text comments in the questionnaire, diaries, etc . | Page 17, row 372-374 |
| - Why were there 3 rounds of assessment for the content validity? This should be made explicit | Thank you, we have now explained the reason for the 3 rounds. | Page 14, row 299-301 |
| - Overall, the method section is hard to follow based on multiple methods for multiples objectives. | The method section is restructured, and headings have been revised in accordance with the two steps/ objectives.  The titles for Figures 3 and 4 are rephrased, to be in line with objective 2.  We have also revised Figure 1 accordingly. | Page 6-18  and  Figure 1 |
| **Results**:  - There is no information on the characteristics of the participants in either step 1 or 2. | See note above about transfer of Table 1. | Page 18, row 406-411. |
| - We believe the sample of n=8 (5 patients and 3 physiotherapists) is very small to properly assess feasibility. This should be addressed (explained) or at least mentioned as a limit of the study. | Thank you, we agree this should be commented on. It was mentioned in the Methodological considerations before, but we have now addressed this matter in more detail in the Limitations section. | Page 31, row 604-609  Page 35, row 699-708 |
| - Table 2, in its current form, is very hard to interpret for the reader. Maybe too much information is presented… | Thank you for pointing this out.  We have now added more descriptive text to the Table 2 title and re-formatted the table columns to make reading more intuitive for the reader. | Page 22-24. Table 2- revised |
| **Discussion**:  - The discussion is well written and supported by the literature. However, the interpretation of the results by the authors should be much more nuanced considering the limits of the study. We urge the authors to add a proper section on the limits and strength of the study, including sample size | We have now added a ‘Strengths and limitations’ section | Page 35, row 692-708 |
| Based on these observations, my opinion is to recommend major revisions of the manuscript. The major flaws include the poor congruence between the objectives and the described methodology, which is hard to follow. We suggest to the authors to divide more clearly the different objectives pursued in the article. Also, we believe that there is important information lacking in the description of the methodology concerning aspects such as the recruitment process of the participants, definition of subject experts, the lack of transparency of the consensus process, the data analysis of qualitative data and a more nuanced interpretation of the results based on the limits highlighted. In its current form, there are major bias that could be addressed by a more structured and detailed methodology. Moreover, we are wondering if the proposed article is too big in its current form and whether it should be preferable to focus on content validity and feasibility in 2 separate articles. | Thank you for pointing at these important gaps in our manuscript - we have now re-structured and detailed more on the methodology and we have also addressed the study limitations. |  |
