# Supplementary material for: Evaluation of content validity and feasibility of the eVISualisation of physical activity and pain (eVIS) intervention for patients with chronic pain participating in interdisciplinary pain rehabilitation programs
Source: PLoS One. 2023 Mar 10;18(3):e0282780. doi: 10.1371/journal.pone.0282780 (PMC10004540; doi:10.1371/journal.pone.0282780)
Supplement: Attachment — Submitted filename: Response to Reviewers.docx [file pone.0282780.s002.docx]

**Reviewer Comments - point-by-point response**.
*Please note that indicated pages and lines refer to the revised paper without tracked changes, the file labeled 'Manuscript'.*

**Reviewer #1:**
Dear Authors,
I have no further considerations about this manuscript. You have done a good job on this review.
Best Regards
**Author response**
Thank you for your kind response. We are grateful for the time and effort you have put into evaluating our manuscript and your provided guidance for improvement.

**Reviewer #3:**
Thank you for the opportunity to review your manuscript. You have undertaken a study to evaluate the content validity and feasibility of your eVIS intervention prior to undertaking an effectiveness trial.

This is a very comprehensive description of the work you’ve done to test and improve your intervention, in both a pre-clinical and a clinical setting. Your methods and results are quite clear, and I feel you have addressed the detail and flow issues raised by the previous reviews. I only have a few very minor points for your consideration.

Minor comments:

**1. Page 5, Line 83.** You seem to have a duplicated sentence here.
**Author response**
Thank you for noticing. We have now deleted the duplicated sentence. *Page 5, Line 83.*

**2. Page 5, line 90.** “activity prescriptions based on individual hinders…”. I’m not sure what a ‘hinder’ is. Do you mean barrier? You may wish to change this word so it is consistent with the rest of your text.
**Author response**
Thank you for your suggestion, of course it should be “barrier”. We have now replaced the word hinders with barriers. *Page 5, line 88*.

**3. Page 11, line 226**. You have noted here that “further expert interviews were needed regarding two areas emerging as incomplete in previous assessment processes” Are you able to mention here what these two areas were here? Was it related to the pharmaceutical issues?
**Author response**
Thank you, this is a good point. We have now clarified which the two areas were: recruitment logistics and pharmaceutical report function. *Page 11, line 225.*

**4. Table 1** – you have put xxx and x in the table. Can you add a description of what the different number of x’s mean in the table legend? I assume it’s the number of rounds they participated in?
**Author response**
Thank you for your kind suggestions for improvement. We have now extended the table legend for a clearer explication of what is detailed in the table. We have also added a symbol explanation of the “x” in the footnote. *Page 18-19, Table 1, line 2 and 415.*

THANK YOU!
